# A Novel Approach for Developing Efficient and Convenient Short Assessments to Approximate a Long Assessment

**DOI:** 10.3758/s13428-021-01771-7

**Published:** 2022-01-31

**Authors:** Yuan Hong Sun, Hong Luo, Kang Lee

**Affiliations:** 1 Hangzhou Normal University Affiliated Hospital, Zhejiang, Hangzhou China; 2 Faculty of Applied Science & Engineering, University of Toronto, Toronto, ON Canada; 3 Institute of Child Study, University of Toronto, Toronto, ON Canada

**Keywords:** machine learning, assessment, shorten, the Long to Short approach, questionnaire, survey, anxiety, depression, stress, anxiety disorder, mood disorder

## Abstract

This paper describes a novel Long to Short approach that uses machine learning to develop efficient and convenient short assessments to approximate a long assessment. This approach is applicable to any assessments used to assess people’s behaviors, opinions, attitudes, mental and physical states, traits, aptitudes, abilities, and mastery of a subject matter. We demonstrated the Long to Short approach on the Depression Anxiety Stress Scale (DASS-42) for assessing anxiety levels in adults. We first obtained data for the original assessment from a large sample of participants. We then derived the total scores from participants’ responses to all items of the long assessment as the ground truths. Next, we used feature selection techniques to select participants’ responses to a subset of items of the long assessment to predict the ground truths accurately. We then trained machine learning models that uses the minimal number of items needed to achieve the prediction accuracy similar to that when the responses to all items of the whole long assessment are used. We generated all possible combinations of minimal number of items to create multiple short assessments of similar predictive accuracies for use if the short assessment is to be done repeatedly. Finally, we implemented the short anxiety assessments in a web application for convenient use with any future participant of the assessment.

## Introduction

Many forms of assessments and surveys are used to measure people’s abilities, aptitudes, attitudes, opinions, skills as well as mastery of academic topics, and overall wellbeing. These assessments and surveys often consist of many items and can take anywhere from several minutes to several hours to complete. Such tools, when properly constructed and validated, can generally provide reliable and valid measurements of what they are designed to assess if the participants respond to all items. However, participants may not have time or patience to complete all items for various reasons, resulting in either fewer participants being willing to complete the full assessment or survey or poor measurement quality if the participants are forced to complete it.

To address this problem, here we propose a novel Long to Short approach that uses machine learning to develop efficient and convenient short assessments or survey to approximate an equivalent long one with acceptable accuracies. The Long to Short approach is based on the fact that (1) items in many assessment and surveys often correlate with each other and thus obtain similar information from the participants to a varying degree (Bakk et al., 2013; Haroz et al., 2020; Lovibond & Lovibond, 1995), and (2) machine learning is able to obtain non-linear combinations of participants’ responses to different items to maximize information needed from the participants (Chatterjee et al., 2014; König et al., 2020; Pintelas et al., 2018). Existing studies have used various non-machine learning techniques such as generalizability theory and item response theory to decrease the number of items required for an assessment or survey (Bakk et al., 2013; König et al., 2020). Generalizability theory has been used to determine the reliability or reproducibility of measurements under specific conditions and thus determine the length of an assessment. Modern test theories such as multidimensional latent class modelling have also demonstrated that one can use the correlations among tested skills to shorten the test length (Haroz et al., 2020). We hypothesized that by capitalizing on these facts, it is possible to reduce the number of items needed to assess participants to achieve an acceptable level of measurement performance (e.g., reliability and validity) similar to that when all items of an assessment are used.

As a proof of concept in this study, here we demonstrate the Long to Short approach by applying it on the assessment of anxiety levels in adults. Anxiety is one of the most common mental health issues affecting the world today. Anxiety is the feeling of worry and uneasiness, usually before important or exciting events. It is a normal human reaction to stressful situations, a fight-or-flight response inherited from human ancestors (Bystritsky et al., 2013). While having a moderated level of anxiety is considered healthy, excessively high levels of anxiety can cause great distress and interfere with daily activities such as work performance and interpersonal relationships (Bystritsky et al., 2013). Sustained exposure to high anxiety levels can eventually lead to anxiety disorders. Anxiety disorders, especially combined with other mental conditions such as major depressive order, can result in poor physical health, moderate to severe psychological distress, and disability (Bystritsky et al., 2013). Anxiety disorders thus put a strain on mental health and healthcare resources, as well as the general wellness of the population (Bystritsky et al., 2013).

Due to the negative effects of high anxiety levels on individuals and the society at large (Wittchen, 2002), there is a worldwide need for regular assessment of anxiety levels, which is mainly provided by mental health professionals. Currently in many parts of the world, mental health resources are insufficient and underfunded to meet this need (Pelletier et al., 2017). Thus, an efficient and convenient tool is urgently needed to help individuals self-monitor their own anxiety levels regularly not only to increase awareness of one’s mental health status but also to prevent the development of anxiety disorders, which in turn lessens healthcare costs and the burden on professional services.

According to the American Society of Clinical Psychology, there are various scales for assessing anxiety symptoms in adults (Mughal et al., 2020). They involve individuals answering a questionnaire, where the response for each question corresponds to a numerical value. Once complete, the values for all the questions are added up to obtain a final score, which can be used to determine the level or severity of anxiety and whether an individual’s anxiety level is above or below a clinical cut-off. The questionnaires can be self-administered or be given by a psychologist or physician (Mughal et al., 2020).

There are several potential issues with existing anxiety assessment tools. First, some of them (e.g., Hamilton Anxiety Ratings Scale (HARS), Hospital Anxiety and Depression Scale (HADS)) must be administered by a physician or clinician, which can be costly and inconvenient. Second, they require answering a somewhat lengthy questionnaire and calculating a score to determine the severity of anxiety levels or the presence of anxiety disorders (Balzer & Schneier, 2006). Some people may not have sufficient time or the patience to complete the whole questionnaire, not to mention that they must consult a manual to score their responses to obtain a final score. Third, the existing anxiety scales tend to assume that each item of a scale carries equal amount of information about the anxiety level of an individual. This assumption is likely to be false (Balzer & Schneier, 2006). Fourth, an individual’s anxiety level needs to be monitored regularly as it changes regularly in response to the changing external or internal factors. Although some people may be willing to complete such a questionnaire once in awhile, its regular use may challenge their patience. In addition, the repeated use of the same questions may lead to memory of one’s previous responses and the re-use of such response, which undermines the assessment’s objective of assessing an individual’s here and now anxiety level. Thus, for effective regular monitoring, the assessment needs to be shortened (Haroz et al., 2020).

The present study aims to determine whether an anxiety assessment scale can be substantially reduced in length but still provide a level of accuracy similar to that when the full scale is used, using the Long to Short approach. To achieve this goal, we used machine learning to identify the optimal subsets of a full anxiety assessment.

Although machine learning has never been used to determine individuals’ anxiety levels, it has been successfully used to classify individuals who have been clinically diagnosed with or without anxiety disorders with reasonable accuracies (Pintelas et al., 2018). These studies have used various types of data, including surveys, clinical notes, physiological data, and even tweets and Facebook posts (Pintelas et al., 2018). For example, Chatterjee et al. (2014) used Logistic Regression, Naïve Bayes, and Bayesian Network techniques on a dataset of heart rates for predicting Generalized Anxiety Disorder, with the Bayesian Network model achieving a 73.33% accuracy for predicting Generalized Anxiety Disorder. Hilbert et al. (2017) implemented a Support Vector Machine (SVM) within a nested leave-one-out cross-validation framework, using a sample of 47 participants (19 with Generalized Anxiety Disorder) and a dataset of clinical questionnaires, cortisol release, gray matter volume, and white matter volume to predict Generalized Anxiety Disorder. They achieved over 90% accuracy to differentiate subjects with a disorder from healthy subjects and 67.46% to differentiate anxiety from major depression. Kessler et al. (R.C. Kessler et al., 2014) used a system of penalized regression, Random Forest (RF), and ensemble machine learning methods to train on a dataset of 47,466 surveys of traumatic experiences exposures from 24 countries. They achieved an area under curve (AUC) scores of 0.96 to 0.98 to predict post-traumatic stress disorder (PTSD). Other machine learning techniques have also been used including gradient boosting techniques such as XGBoost and Gaussian Naïve Bayes (GNB). Feature selection and engineering techniques was also heavily utilized. However, most of the existing studies only focused on predicting anxiety disorders using machine learning techniques. No study has yet applied the machine learning approach to the assessment of anxiety levels, which is one of the major tasks of the present proof-of-concept study.

Our goal here is to develop a set of shortened assessments, or the Rapid Anxiety Assessment, to assess anxiety levels that approximates the results of a longer assessment. To this end, we selected Depression Anxiety Stress Scale 42 (DASS-42), which is a 42-item self-assessment scale for measuring depression, anxiety, and stress, with 14 items for each condition. Each item consists of a statement regarding the participant’s feeling or experience, and the participant can select how closely they match the statement in the past week, with options “never”, “sometimes”, “often”, and “almost always”, which correspond to scores 0, 1, 2, and 3, respectively (Lovibond & Lovibond, 1995). The total score for each condition (e.g., anxiety) is calculated by summing up the scores of all 14 questions corresponding to each condition. Based on the scores for each condition, the severity of the condition can be classified into “none”, “mild”, “moderate”, “severe”, and “extremely severe” levels, based on threshold scores for each condition at each level (see http://www2.psy.unsw.edu.au/groups/dass/ for the complete DASS-42 questionnaire and scoring) (Lovibond & Lovibond, 1995).

We obtained an online DASS survey dataset from a large sample of participants worldwide (N = 31,715). No other sources of data were used. We scored the anxiety levels of each participant according to the DASS manual. DASS-42 uses only the 14 items on the anxiety subscale to derive a final anxiety score which is then categorized into five levels: “none”, “mild”, “moderate”, “severe”, and “extremely severe”. Although the machine learning approach can be used to train computational models to classify participants into these five levels, for simplicity as a proof-of-concept, here we only trained models to classify participants into two categories (“none” and “mild” vs. “moderate”, “severe”, and “extremely severe”; henceforth referred to as the low vs. high anxiety classes). The “moderate” threshold was chosen because individuals scoring at this level and above may need to be alerted and perhaps even advised to seek professional consultation.

Following the standard machine learning practice, we first performed data pre-processing, re-balancing, and argumentation. Next, feature selection was done to select the questions that were best to predict whether participants’ final anxiety scores were at the low or high class. To this end, we used the Minimum Redundancy Maximum Relevance (MRMR) method and extracted the Gini importance of each feature (i.e., question) from an Extra Tree Classifier (Menze et al., 2009) (sklearn.ensemble.ExtraTreesClassifier, 2020). The feature selection was performed on participants’ answers to all 42 questions on DASS even though the scale itself only used 14 questions to compute the final anxiety score and level. We did so because evidence showed that in cognitive measures that contain multiple sub-scales for testing the same target, such as DASS, the items are not necessarily locally independent (Haroz et al., 2020). This means that in DASS, some questions designed to assess stress and depression contained important information about participants’ final anxiety score. Then, different machine learning techniques were used on combinations of items selected from the most important items either with or without demographics information (age, gender, region).

Based on existing studies of predicting anxiety disorders using machine learning techniques, we chose the following techniques in our study:

- Logistic Regression (LR): A statistical model using a logistic function to model a categorical variable, commonly a binary dependant variable (Peng et al., 2002).
- Gaussian Naïve Bayes (GNB): Probabilistic model using a naïve assumption of Bayesian random variables, assuming a Gaussian distribution (Zhang, 2004).
- Support Vector Machine (SVM): A clustering technique that applies the statistics of support vectors to categorize unlabeled data, by deciding sets of hyperplanes that separate different classifications (Evgeniou & Pontil, 2001).
- Random Forest (RF): An ensemble learning method for classification, regression and other tasks that operate by constructing a multitude of decision trees at training time and outputting the class that is the mode of the classes (classification) or mean prediction (regression) of the individual trees (Cutler et. al., 2011).
- Multilayer Perceptron (MLP) neural network: A type of feedforward artificial neural network (ANN) that is composed of multiple layers of nodes with biases and activation thresholds and edges with weights (Wilson & Tufts, 1994).
- Extreme Gradient Boosting decision trees (XGBoost): A variation of the gradient boosting technique, designed to increase system performance. Combines decision trees with Stochastic Gradient Boosting with Regularized Gradient Boosting (Chen & Guestrin, 2016).
- Stacked Generalization Ensemble (Ensemble): A stacking ensemble of various sub-classifiers (GNB, LR, SVM, RF, MLP, XGBoost) using a Logistic Regression classifier (Wolpert, 1992).

To evaluate the performance of each model, per machine learning convention, the Area Under Curve (AUC) of the Receiver Operating Characteristic (ROC) curve score, the F1 score, as well as Precision and Recall scores were used. By doing so, we then compared the performances of the five machine learning techniques to determine the best technique to use shortened assessments to predict whether participants have low or high anxiety levels. In the end, the best performing models based on the best machine learning technique as measured by the above metrics were selected for the implementation on a web application for the Rapid Anxiety Assessment.

We hypothesized that the full DASS scale has items with redundant information about a participant’s anxiety level and thus can be substantially shortened such that the shortened assessment with a subset of items can achieve a similar accuracy to that when the full scale is used. This hypothesis was based on the fact that the developers of the DASS-42 have successfully used the traditional psychometric methods to reduce the scale to 21 items with high concordance validity with the DASS-42 (Lovibond & Lovibond, 1995). The shortened version, DASS-21, achieved a validity of 94% relative to DASS-42 (Henry & Crawford, 2005). Thus, the machine-learning approach, by using non-linear combinations of items might reduce the number of items needed to achieve as high a level of accuracy as the full scale.

We also hypothesized that some of the items of the DASS-42 that do not belong to the anxiety scale might still carry important information about a participant’s anxiety level. Thus, such items might be useful to make accurate predictions about an individual’s anxiety. Furthermore, we hypothesized that demographics information (e.g., age, gender, region of residence) about the user can also contain information about an individual’s anxiety status, since research have shown that demographic differences can affect the severity and presentation of anxiety symptoms (Faravelli et al., 2013).

Regarding specific machine learning methods, we hypothesized that non-linear combinations of the most important items should provide additional accuracy boost to the shortened assessments and thus ensemble machine learning methods such as XGBoost and Stacked Generalization Ensemble should perform significantly better than the traditional machine learning techniques such as SVM and Logistic Regression.

## Methods

### Participants

The dataset used for this study contained a total of 31,715 participants from around the world. Among them, 7,217 were males and 24,498 were females. The mean age of participants was 25.4 years, and the standard deviation was 23.7. The largest demographic by far was young adults aged 18-27 years, consisting of 24,673 participants. There were progressively less participants with increased age. In terms of the country of residence, a large majority came from Asia, consisting of 22,412 participants. There were 6,006 participants from North America, 2,498 from Europe, 623 from Oceania, 245 from South America, and 201 from Africa. A detailed breakdown of the demographics by continent is shown in Table 1.

**Table 1:** Breakdown of demographics by continent

Continent	Asia	Europe	North America	Other
Mean Age (years)	23.6	28.5	29.9	28.7
Standard deviation Age (years)	27.0	11.3	12.9	11.6
Males	3,896	955	2,016	350
Females	18,246	1,543	3,990	719

In terms of demographics, this dataset was quite imbalanced by gender, age, and region. There were many more females than males because females are generally more interested about their psychological or mental wellbeing compared to males. Since the original survey was online (which will be described in detail below), it was more accessible by young adults, who are generally more well-versed with technology; thus, the majority of respondents were young adults. Finally, because the population of Asia is much greater than other continents, there were many more participants of this survey from Asia than from other continents. The number of participants from each continent were roughly proportional to their populations.

### Materials

For this study, we used Depression Anxiety Stress Scale (DASS). DASS was developed by the University of New South Wales, Australia in 1995. It is available in two versions, DASS-21, and DASS-42, where they differ by the total number of items in the questionnaire (21 and 42 respectively). This study uses DASS-42, which is a 42-item self-report scale designed to measure the three negative emotional states of depression, anxiety, and stress. There are 14 items to measure each condition. For each item, a statement regarding the participant’s feeling or experience is given, and the participant can answer how closely they match the statement in the past week, with options “never”, “sometimes”, “often”, and “almost always”, which correspond to scores 0, 1, 2, and 3, respectively (Lovibond & Lovibond, 1995). The total score for each condition (e.g., anxiety) is calculated by summing up the scores of all 14 questions corresponding to each condition (see http://www2.psy.unsw.edu.au/groups/dass/Download%20files/Dass42.pdf for the complete DASS-42 questionnaire). Based on the scores for each condition, the severity of the condition can be classified into “none”, “mild”, “moderate”, “severe”, and “extremely severe” categories, based on threshold scores for each condition at each level (for more information, please see the DASS manual at http://www2.psy.unsw.edu.au/groups/dass/) (Lovibond & Lovibond, 1995). For the present study, we combined the none and mild into one class (low anxiety) and the rest into another (high anxiety).

The DASS scale was developed using a sample of responses from 504 sets of students. It was then normalized on a normative sample of 1,044 males and 1,870 females aged between 17 and 69 years, coming from various backgrounds. The scores were subsequently validated against multiple outpatient groups including patients suffering from anxiety, stress, and depressive disorders, as well as other mental disorders (Lovibond & Lovibond, 1995). The reliability scores of the scales in terms of Cronbach's alpha scores, or the tau-equivalent reliability, rate the Depression scale at 0.91, the Anxiety scale at 0.84, and the Stress scale at 0.90 in the normative sample (Lovibond & Lovibond, 1995). The present study only focuses on adults aged 18 and above; however, according to the DASS manual, although DASS is only validated on adults, due to the simplicity of language, it can be reliably used in children as young as 12.

### Participant Data Collection Procedure

The data was collected for this survey by posting an online survey accessible worldwide. The survey was anonymous, where the users were consented to provide their personal information and signed a code of ethics. The survey included all 42 questions in DASS-42, as well as a series of questions that asked for the demographics of the participant. The survey entries were then downloaded and organized into a comma separated values (CSV) format as the main dataset for this study.

The dataset included answers from all 42 items of the DASS-42 questionnaire, the participants’ demographics information including gender (not biological sex), age at the time of survey, and country of residence at the time of survey. It also included the anxiety, depression, and stress scores calculated from the DASS-42 scoring method, as well as the times taken for each question to track if the survey was properly answered. The answer values for each option for every DASS item were encoded numerically using integers 0, 1, 2, and 3, representing the options “never”, “sometimes”, “often”, and “almost always” in the questionnaire, respectively. Additional materials included the severity classification based on the scores for each of the three conditions. Since this study only focuses on anxiety, the depression and stress scores were discarded.

The DASS-42 questionnaire defines threshold scores for every severity level of anxiety (“none”, “mild”, “moderate”, “severe”, and “extremely severe”). However, for the present study, we classified participants into two anxiety levels according to their responses and scores: If the DASS anxiety score was in the category of “moderate”, “severe”, and “extremely severe”, we assigned the participants into the class of high anxiety levels (1); if the DASS anxiety score was in the category of “none” or “mild”, we assigned the participants into the class of low anxiety levels (0). Using this criterion, we obtained 20,849 high anxiety samples and 10,866 low anxiety samples. This is a quite unbalanced dataset, which would be later dealt with in the data preprocessing.

In the DASS-42 questionnaire, there are 14 questions for each of depression, anxiety, and stress scales. For this study, however, the questions related to stress and depression would not be removed. All 42 questions from DASS were taken into consideration as features for feature selection and training so that the machine learning techniques could be applied to assess which questions would be more important for optimally predicting anxiety.

**Fig. 1:**
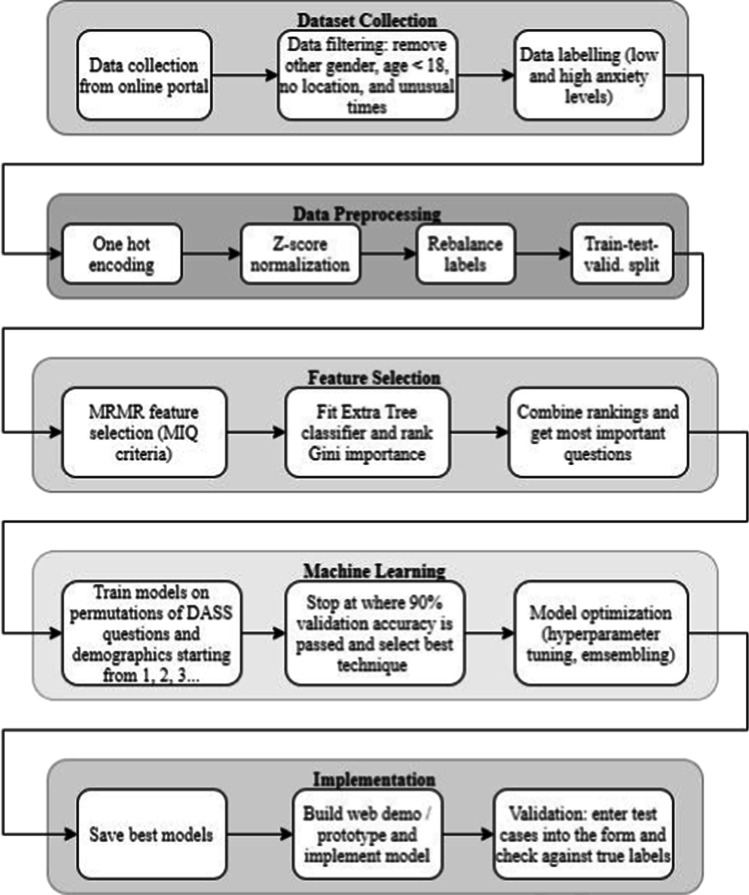
Flowchart description of the data analysis methods used for this study

The dataset also includes various demographics features, including age, gender, and country of residence. Age is an integer of the participant’s age when the survey was completed. Gender is an integer where different values represent male (1) and female (0). Country is a two-character country code (e.g., CA – Canada, US – United States). For simplicity, it was re-organized into 3 regions, “East”, “West”, and “Other”. “East” consists of countries in Asia, “West” consists of Europe, North America, and Oceania, while “Other” encompasses the remainder of the countries. All the other demographics fields in the dataset, such as marital status, family size, religion, and education level, were not used for the present proof-of-concept study, although they could be used in future studies to improve prediction performance Fig. 1.

### Data Analysis

Below is a workflow description of the data analysis.

#### Data Preprocessing

For this study, the original dataset was filtered based on specific conditions. First, since this study focuses on adults only, rows where age was smaller than 18 were removed. Second, rows where country or region of residence was empty were also removed. Third, rows with unusual times taken for each question (e.g., too short, or too long: 2 standard deviations from the mean) were also removed.

After filtering the data, first, the raw data features were organized and processed. All the columns that could be categorized in the dataset were transformed into one-hot encoding representing each category. For instance, gender could be transformed into two columns representing each gender, and only one column could be marked as 1 for every row (while the other columns remained as 0). The transformed columns included the answers to all multiple-choice questions in the DASS-42 questionnaire (answers range from integers 0 to 3, representing the options “never”, “sometimes”, “often”, and “almost always”), gender (1 for male and 0 for female), and regions (2 for “East”, 1 for “West”, and 0 for “Other”). This was also applied to the label column for anxiety status, where one column indicated whether the sample is of high (1) or low (0) anxiety status.

All the scalar features, such as age, were then normalized using the z-score method:

$$Z=\frac{X-\mu }{\sigma }$$

where *x* is the sample, μ is the population mean, and σ is the population standard deviation (Kreyszig, 1979).

Next, after checking the counts of rows with each label, the dataset was balanced by up sampling the low anxiety samples to match up with the number of high anxiety samples. Prior to the up sampling, there were 10,866 low anxiety samples and 20,849 high anxiety samples. The up-sampling algorithm randomly selected rows from the specified categories and duplicated them in the dataset, so the row count matched the other categories (Sklearn.utils.resample, 2020). This was critical to ensure that the dataset was not biased towards a certain category, thus affecting the model results. After re-balancing, there were 20,849 × 2 = 41,698 samples in total in the dataset.

Finally, the features and labels were separated, and the dataset was partitioned into training, testing, and pristine validation datasets. In this study, 80% of the dataset (33,358 samples) was to train the models, 10% (4,170 samples) of the dataset was used for internal model testing during training, and the remainder 10% (4,170 samples) was held out and used for model external validation after completing the training. Throughout the training process, the training and internal test sets were reshuffled and re-partitioned to ensure model consistency. The pristine external validation set was never used for training and testing. Each model’s performance was then evaluated against the pristine external validation set. This ensured our findings to be generalizable to a new dataset.

#### Feature Selection

Since the goal was to reduce the number of questions required to accurately predict anxiety levels, the first step of the Long to Short approach was to use feature selection techniques to check which questions from the DASS-42 questionnaire weighed more heavily in predicting low vs. high anxiety levels. This was completed on the full processed and balanced dataset (41,698 samples).

We first applied the Minimum Redundancy Maximum Relevance (MRMR) feature selection technique, using the Mutual Information Quotient (MIQ) criteria, to identify the most important questions used to predict anxiety from the DASS-42 questionnaire. As mentioned earlier, all 42 DASS-42 questions were included in the pool because some questions designed to assess stress and depression in DASS might contain important information about participants’ final anxiety score. MRMR is an unsupervised technique, and it selects the most relevant features based on pairwise correlations, or mutual information of each pair of variables in the dataset, while minimizing the redundancy between variables (Peng et al., 2005).

To serve as a reference, a second supervised feature selection method was devised by fitting an Extra Tree Classifier for all the features and labels before ranking the most important features. This step was to ensure the robustness of the feature selection by MRMR, and to validate the results. The Extra Tree classifier fits several randomized decision trees (a.k.a. Extra Trees) on sub-samples of the dataset and averages the results. The feature importance was obtained by computing the normalized total reduction of the criterion brought by that feature, which is known as the Gini Importance (sklearn.ensemble.ExtraTreesClassifier, 2020). Gini Importance, also known as Mean Decrease in Impurity (MDI), calculates each feature importance as the sum over the number of splits across all decision trees that include the feature, proportionally to the number of samples it splits (Menze et al., 2009). The ranking of the most important questions from DASS-42 were obtained by ranking the feature importance.

Finally, we combined the most important questions from DASS-42 from both the MRMR and the Extra Tree classifier. The top 10 most important questions from DASS-42, along with the demographics features of age, gender, and region were then selected to form the pool of features that would be used in the machine learning model training. Although more DASS-42 questions could have been selected, only the top 10 DASS-42 questions were used as this saved the overall computational time. Our goal was to select a subset of questions from the 10 questions and to use the selected questions to train computational models to predict participants’ anxiety levels. To do so, we had a choice of a minimal 1 question to a maximal of 10 questions, and thus, there were 1,023 possible combinations to test, which already took substantial computational time. Further increase in the number of questions to be tested would lead to an exponential increase in computational time, as shown by the formula below:

$$Total\ combinations\ of\ n\ items={\sum}_{k=0}^n\left(\genfrac{}{}{0pt}{}{n}{k}\right)={2}^n-1$$

Because the present research was a proof-of-concept study, we limited the largest number of DASS-42 questions to be 10.

#### Model Training

The second step of the Long to Short approach was to train the machine learning models that predict low vs. high anxiety levels, based on combinations of the top 10 DASS-42 questions identified from the feature selection, as well as demographic features in the dataset including age, gender, and region. The main purpose of this was to find the minimum number of questions from DASS-42 that would be required to compose a sufficiently accurate assessment for predicting anxiety levels. To do so, we started off by randomly selecting one question from the 10-question pool to train computational models to classify participants into the low vs. high anxiety classes. We evaluated the model performance to determine whether 1 question plus demographics was sufficient to classify participants accurately. After training with 1 out of 10 questions, we moved onto training models with combinations of 2 out of 10 questions, again by randomly selecting 2 questions from the 10-question pool. We then increased the number of questions to 3, 4, etc. until 10 and performed machine learning to produce computational models to classify participants based on 3, 4, …10 DASS-42 questions plus the demographics. We compared the results with and without 3 demographics items (gender, age, and region of residence) to determine their contributions to model performances. As a result, we obtained two sets of machine learning models, one with and the other without demographics as features.

We did not experiment with more than 10 questions as the computation time would be too much for this proof-of-concept study. For every number of DASS-42 questions, we ran 10 different combinations of DASS questions from the pool of top 10 questions. We did not run more than 10 combinations as we simply wanted to observe a general trend in this study, and the computation time would increase with each added combination. Additionally, the combinations were kept the same across all models, for every number of questions, for a fair comparison between models.

For each model training, as mentioned earlier, we split the dataset into training (80%), test (10%), and pristine validation datasets (10%). The machine learning training was done on the training dataset only, and internally validated on the test dataset. The label or target was a binary column representing the level of anxiety (0 for low and 1 for high anxiety level). After we obtained one model, we recombined the training and testing datasets and partitioned them in the same way (80% vs. 10%) and trained and tested another model. We did data recombination, partitioning, and training/testing 50 times because our preliminary training revealed that 50 sub-models was sufficient to produce a stable Gaussian distribution of model performance metrics (see below). We then tested the 50 sub-models to classify participants in the holdout validation dataset to assess how well these models performed (see below). The holdout validation dataset, which contained 10% of the complete dataset, was untouched during the entire process. This made it a fair comparison between models since it represented the accuracy when models are used for classifying individuals whose data had never been used in training and testing. In other words, the results from the pristine holdout data represented how well the models would perform when used with real-world users.

The techniques tested in this study included Logistic Regression, Gaussian Naïve Bayes, Support Vector Machine (SVM), Random Forest (RF), Multilayer Perceptron neural network (MLP), XGBoost (Extreme Gradient Boosting decision trees), and Stacked Generalization Ensemble. The main loss function used in machine learning was the Binary Cross-Entropy (BCE) loss. All the machine learning techniques had been implemented in Python 3 using its Scikit-learn libraries and were kept in their default hyperparameters and stopping criteria for a fair comparison. The hyperparameters and stopping criteria are listed in Appendix A.

The primary loss function used to evaluate the model during training was the Binary Cross-Entropy (BCE) loss, which measures the average logarithmic difference between the predicted values (*p*(*y*_*i*_)) and the actual value (*y*_*i*_) in a binary classifier (BCELoss., 2020).

$${H}_p(q)=\frac{1}{N}\sum_{i=1}^N{y}_i\log \left(p\left({y}_i\right)\right)+\left(1-{y}_i\right)\log \left(1-p\left({y}_i\right)\right)$$

For each model, the following metrics were evaluated on the holdout validation dataset:

- Area Under Curve (AUC) score of Receiver Operating Characteristic (ROC) curve: Measures the diagnostic ability of a binary classifier system by plotting the True Positive Rate (TPR) against the False Positive Rate (FPR) at various threshold settings. The ROC is a probability curve, and the AUC represents the degree of separability (Bradley, 1997).
- Precision: Number of true positives (TP) over the number of true positives (TP) plus the number of false positives (FP) (Precision-Recall., 2020).
  $$Precision=\frac{TP}{TP+ FP}$$
- Recall (Sensitivity): Defined as the number of true positives (TP) over the number of true positives (TP) plus the number of false negatives (FN) (Precision-Recall., 2020).
  $$Recall=\frac{TP}{TP+ FN}$$
- F1 Score: Harmonic mean of Precision and Recall, measures the performance of a model’s classification ability over both positive and negative cases (Precision-Recall., 2020).
  $$F1=2\times \frac{Precision\times Recall}{Precision+ Recall}=\frac{TP}{TP+\frac{1}{2}\left( FP+ FN\right)}$$

The above metrics were taken on all 50 sub-models for each combination of DASS questions and demographics, across 10 combinations for every number of questions from DASS, from 1 until whenever the accuracy was sufficient. The average across the 10 combinations was taken, as well as the 95% confidence interval across 10 combinations.

#### Model optimization

The next step of the machine learning was to optimize the hyperparameters for the best performing models. This was done so that the best machine learning models from the above five techniques could be further improved. We first chose the best performing models based on the AUC ROC score and F1 score evaluated against the pristine validation dataset. The best models were re-trained using the same procedure from above, and the best hyperparameters for each model were obtained by using a grid search, where different combinations of hyperparameter values were used to train each iteration to determine which combinations are the best. The best models at the end were chosen for implementation into the Rapid Anxiety Assessment tool.

#### Model Implementation

The best machine learning models developed from the previous step were implemented in the prototype of the Rapid Anxiety Assessment tool so they could be validated. The validation ensured that the models were implemented correctly. The validation consisted of two steps. The first step was by entering a fixed set of responses, with permutations of different values for different demographics and DASS items, for all sets of the questionnaire and checking if the predicted level of anxiety was realistic. It would simulate different combinations of responses that a user would input into the tool. The second step was to enter several different responses from a pristine dataset and tallying the average binary prediction accuracy (AUC ROC score and F1 score) to match that of the pristine dataset when tested on the raw models, where the numbers must match exactly.

## Results

### Feature Selection

All 42 items, numbered from 1 to 42, from the DASS-42 questionnaire were included in the feature selection process to select the most important items.

Selecting the top 10 most relevant items in DASS-42 using the MRMR method returned the item numbers {11, 30, 6, 13, 14, 18, 29, 27, 39, 20}. Only 2 out of 10 items here {30, 20} were used for calculating the DASS anxiety score. Fitting the Extra Tree Classifier on all features and labels and taking the top 10 most important items by Gini Importance returned the item numbers {34, 11, 13, 23, 17, 15, 12, 19, 2, 33} from DASS-42. 4 out of 10 items here {23, 15, 19, 2} were used for computing the DASS anxiety score; 2 out of 10 items here {11, 13} were also found to be among the 10 most important items from MRMR. Combining the results from MRMR and Extra Tree, the 10 items numbered {11, 13, 34, 23, 17, 15, 12, 4, 2, 33} carried the most importance and were selected for the next stage of analysis.

### Model Training

**Fig. 2:**
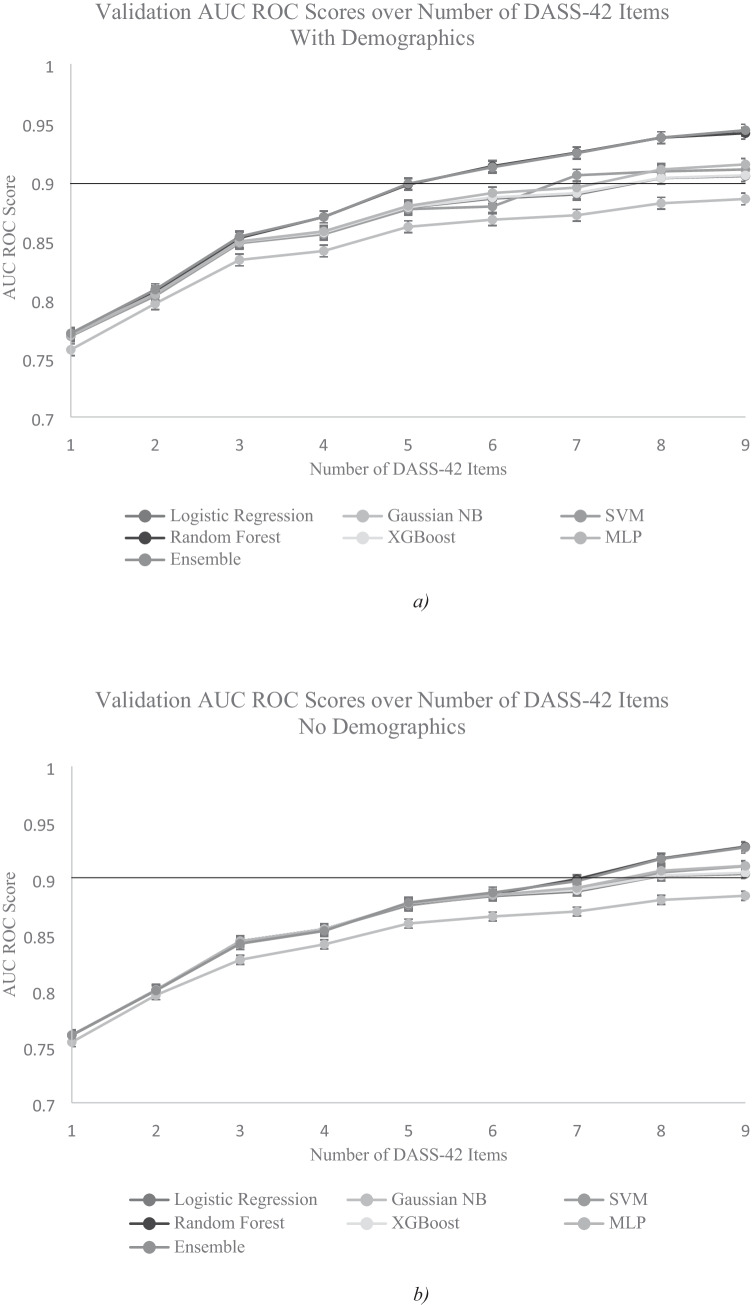
Validation Area Under Curve (AUC) scores of the Receiver Operating Characteristic (ROC) curve for all models, averaged over 10 combinations of different numbers of questions from DASS-42 (n). The total number of questions is (n+3) with demographics and n without demographics. A horizontal line shows the 90% threshold. **a**). With demographics. **b**). Without demographics

After training all the machine learning models, the test results were plotted. Figure 2 show the AUC of ROC scores, for combinations of 1 DASS question through 9 questions, with and without 3 demographics features (age, gender, region), averaged over 50 ensembles / sub-models for each model / combination, over 10 combinations for each number of questions, and across the five machine learning techniques outlined above, on the validation dataset. The error bars represent the range of the 95% confidence interval of each metric over the 10 combinations.

Results revealed that the models performed better as the number of questions in the training features are increased, but the amount of performance improvement diminished with more questions. Figure 2a shows the test AUC ROC scores for the best models exceeded 90% at 5 questions from DASS plus 3 demographics items, which we determined to be the desired accuracy threshold. From Fig. 2b, it took approximately 7 DASS items without demographics for the best models to exceed 90% AUC ROC. Thus, we proposed that a minimum of 5 items from DASS-42 plus 3 demographics items could be used for rapid assessment for the levels of anxiety if the use case allows for collecting demographic data, and 7 items from DASS-42 if the use case does not call for the input of demographics. In addition, it is interesting to note from Fig. 2a and b showed that 5 DASS items with demographics performed significantly better than 5 DASS items without demographics. This result suggested that demographic fields such as age, gender, and region of residence carry notable predictive power about anxiety levels. It took 7 DASS items without demographics to roughly match the accuracy of 5 DASS items with 3 demographics items (8 items total).

A more detailed table of results for all the models run on combinations of 5 DASS items and demographics is presented in Table 2, and combinations of 7 DASS items and no demographics in Table 3. Based on Tables 2 and 3, Ensemble and Random Forest were the best performing models based on the metrics of AUC score and F1 score (also see Figure 2). However, since their 95% confidence intervals overlapped, it was not clear which technique performed better. Because the Ensemble model has more tunable parameters, it was more likely for it to exceed the performance of Random Forest if we adjusted hyperparameters of both models.

**Table 2:** Comparison of validation accuracies of the best models trained on combinations of 5 questions, averaged over 10 combinations, using default hyperparameters, with demographics.

Model	AUC Score	Standard Deviation	95% Confidence Interval	F1 Score	Standard Deviation	95% Confidence Interval
Logistic Regression	87.79%	0.54%	87.58% - 87.97%	87.76%	0.55%	87.55% - 87.95%
Gaussian NB	86.16%	0.41%	86.02% - 86.30%	86.05%	0.42%	85.92% - 86.20%
SVM	87.66%	0.70%	87.40% - 87.93%	87.61%	0.71%	87.35% - 87.89%
MLP	87.93%	0.87%	87.44% - 88.01%	87.89%	0.87%	87.40% - 87.97%
Random Forest	89.75%	0.72%	89.52% - 89.96%	89.67%	0.71%	89.43% - 89.88%
XGBoost	87.82%	0.96%	87.46% - 88.18%	87.80%	0.93%	87.43% - 88.14%
Ensemble	89.82%	0.84%	89.60% - 90.07%	89.78%	0.82%	89.56% - 90.03%

**Table 3:** Comparison of validation accuracies of the best models trained on combinations of 7 questions, averaged over 10 combinations, using default hyperparameters, without demographics

Model	AUC Score	Standard Deviation	95% Confidence Interval	F1 Score	Standard Deviation	95% Confidence Interval
Logistic Regression	88.84%	0.57%	88.61% - 89.04%	88.81%	0.58%	88.58% - 89.01%
Gaussian NB	87.05%	0.45%	86.91% - 87.20%	86.95%	0.45%	86.91% - 87.20%
SVM	89.00%	0.68%	88.79% - 89.22%	88.94%	0.66%	88.73% - 89.15%
MLP	89.16%	0.79%	88.87% - 89.48%	89.11%	0.80%	88.81% - 89.43%
Random Forest	89.94%	0.74%	89.63% - 90.33%	89.88%	0.73%	89.57% - 90.26%
XGBoost	88.97%	0.91%	88.56% - 89.38%	88.93%	0.92%	88.51% - 89.35%
Ensemble	89.77%	0.85%	89.49% - 90.07%	89.73%	0.86%	89.45% - 90.04%

Thus, in the next step, we selected the best performing methods Random Forest and Stacked Ensemble by optimizing their hyperparameters. The best model test results from each of the two techniques are summarized in Table 4.

**Table 4:** Comparison of validation accuracies of best models trained on combinations of 5 questions, averaged over 10 combinations, using optimized hyperparameters, with and without demographics

Model	AUC Score	Standard Deviation	95% CI	F1 Score	Standard Deviation	95% CI	Precision	Recall
With demographics								
Random Forest	90.54%	0.46%	90.38% 90.69%	90.50%	0.47%	90.33% 90.65%	90.48%	90.44%
Ensemble	90.80%	0.51%	90.63% 90.97%	90.77%	0.50%	90.60% 90.95%	90.72%	90.77%
Without demographics								
Random Forest	90.92%	0.51%	90.69% 91.07%	90.87%	0.51%	90.66% 91.04%	90.90%	90.97%
Ensemble	91.51%	0.56%	91.33% 91.68%	91.47%	0.57%	91.30% 91.64%	91.43%	91.48%

We found that when we adjusted hyperparameters, Random Forest did not improve much compared to the baseline model, whereas the results for Ensemble improved noticeably. The 95% confidence interval for AUC and F1 scores of the Ensemble model exceeded the ranges for Random Forest. The Precision and Recall metrics were also significantly in favour of Ensemble. Thus, for the remainder of the study, efforts would be focused on improving the performance of the Stacked Ensemble technique on combinations of 5 questions from the top 10 most relevant questions in DASS-42 and demographics features (age, gender, and region), and implementing the model into the Rapid Anxiety Assessment tool.

Further improvements were made to the Ensemble model by tuning the sub-estimators. The number of sub-models for each model was also reduced from 50 to 10 to improve memory performance, while sacrificing little in terms of model performance. This adjustment resulted in an average validation AUC score of 91.86% and F1 score of 91.83% across 10 combinations of 5 questions. Finally, out of the 10 combinations, the top 5 combinations that produced the highest AUC and F1 scores were chosen for implementation into the Rapid Anxiety Assessment tool. The best combination had a validation AUC score of 92.74% and F1 score of 92.63%. This was a noticeable improvement over the baseline models.

**Fig. 3:**
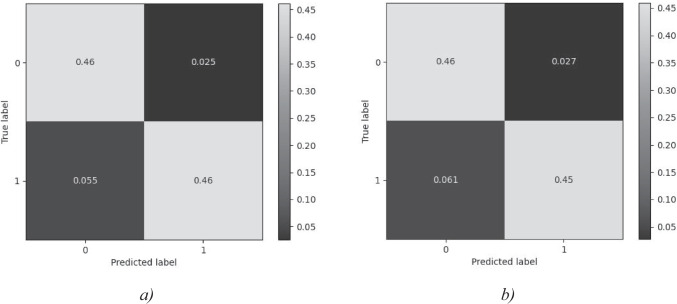
Confusion matrix of the Ensemble model on the validation dataset (0 is low anxiety level and 1 is high anxiety level). **a**). With demographics **b**). Without demographics

**Fig. 4:**
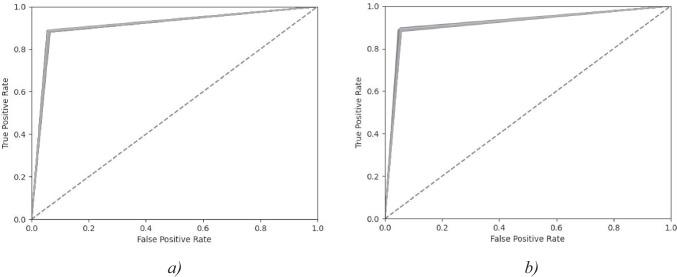
ROC (Receiver Operating Characteristic) curve graph of the best Ensemble model and its sub-models on the validation dataset. The dashed line represents the ROC curve for a random classifier (AUC ROC score = 0.50). **a**). With demographics, **b**) Without demographics

For further examination of the data, the best Ensemble model was taken, with and without demographics, and their confusion matrices and ROC (Receiver Operating Characteristic) curve graphs were plotted for all of the sub- models, shown in Figure 3 and Figure 4. Clearly, this model had high sensitivity and high specificity (i.e., low false alarm rate). From the confusion matrix, this model classified participants most accurately with minimal misclassifications. From the AUC curve, for about 92% of the time, the model could distinguish between low and high anxiety samples.

### Implementation

We built a prototype of the Rapid Anxiety Assessment tool in the form of a web application to showcase its functionality. The demo app allows the user to answer two short surveys. The first one includes age, gender, region, and a set of five questions/items from the DASS-42 questionnaire, a total of 8 items. The second one only has a set of seven items from DASS-42. It takes approximately a minute for the user to complete one survey. Once submitted, the app instantly computes the probability that the user has anxiety using a model in the background, which takes less than a second, and displays the results on a separate results page. The DASS question sets are randomized every time based on which questions the models require, with a total of 5 question sets each. The models implemented use the optimized Stacked Generalization Ensemble technique and are ensembles of 10 different sub-models for every set of questions. There are 5 different sets or versions of questions for each survey, making it 10 sets in total.

**Fig. 5:**
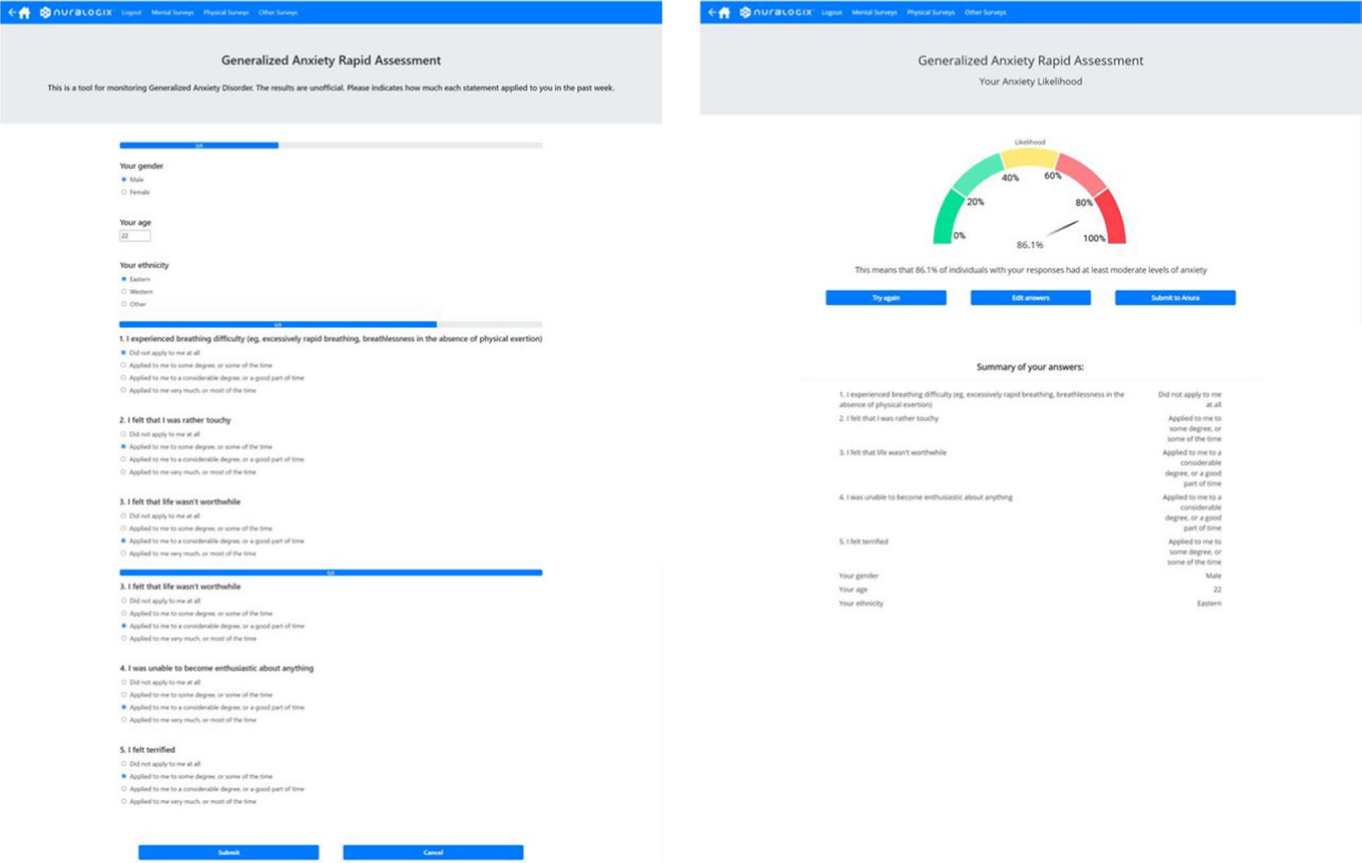
Left: Screenshots of questionnaire page of the demo app for the Rapid Anxiety Assessment. The user completes the questionnaire form of 8 questions in total (3 demographics and 5 DASS questions) and a progress bar keeps track of the number of questions answered. Right: Screenshots of the results and summary pages of the demo app. A dial displays the probability / likelihood of high anxiety level, and the answers are summarized. Full screenshots are available in Appendix C

The application was written in Python 3 and HTML/CSS using a web framework called Flask (https://palletsprojects.com/p/flask/). The current demonstration is an internally hosted prototype for validation purposes. Figure 5 below demonstrates an example usage of the tool. The demo can be found by logging into this website: https://survey-demo.deepaffex.ai/. Instructions on how to access the web demo are listed in Appendix B. Screenshots from the demo are available in Appendix C.

## Discussion

The purpose of this study was to develop a machine-learning-based approach for shortening long questionnaire-based assessments while retaining their accuracy relative to the long assessment. This is known as the Long to Short approach. To show a proof-of-concept of the Long to Short approach, we shortened the DASS-42 scale to predict low vs. high anxiety levels in adults.

Our findings confirmed most of our hypotheses. We hypothesized that the full DASS scale has items with redundant information about a participant’s anxiety level and thus can be substantially shortened such that the shortened assessment with a subset of items can achieve a similar accuracy to that when the full scale is used. We showed that we could reduce the number of questions in a questionnaire-based assessment while retaining a relatively high accuracy. In this study, we achieved over 90% accuracy on a reduced DASS-42 scale for measuring low vs. high levels of anxiety that only has five questions plus demographics or seven questions without demographics. This finding is in line with previous efforts using non-machine learning based methods (Henry & Crawford, 2005; Lovibond & Lovibond, 1995).

We also hypothesized that some of the items of the DASS-42 that do not belong to the anxiety scale might still carry important information about a participant’s anxiety level. Indeed, we found that in the DASS-42 scale, some of the items that do not belong to the anxiety scale itself still carried important information about a participant’s anxiety level. Thus, these items were useful for making accurate predictions about an individual’s anxiety levels. Furthermore, although demographics were originally not a part of the DASS scale, we discovered that it carried significant information about one’s anxiety levels. However, they are not as important as DASS items themselves because it took only 2 additional DASS items (7 in total) to match the accuracy of 5 DASS items and 3 demographics items (8 in total). The assessment with 7 DASS items without demographics may be more suitable for the Rapid Anxiety Assessment tool since it is shorter and does not require the user to enter additional personal information.

Regarding machine learning methods, we hypothesized that more advanced ensemble machine learning methods such as XGBoost and Stacked Generalization Ensemble should perform significantly better than traditional machine learning techniques such as Logistic Regression and Random Forest. This hypothesis was only partially supported. We found that the traditional Logistic Regression and the more advanced techniques performed similarly well to classify participants. In some cases, it performed statistically slightly better than XGBoost and the same as Stacked Ensemble. Only after we tuned the hyper-parameters, did the Stacked Ensemble model’s performance improved significantly more than the baseline after hyperparameter tuning and eventually outperformed Random Forest. These findings suggest that a traditional machine learning technique such as Random Forest may still be a useful technique for analyzing the type of data we have, given its efficiency and lower demand on computational resources.

Overall, our findings taken together show the feasibility of using our Long to Short approach that uses machine learning to train computational models to obtain shorten assessments to approximate the performance of an original long assessment.

As this study was exploratory, it also had a few limitations which should be addressed in the future. First, the sample size of this study, though appearing large, was still relatively small for conducting machine learning training to obtain highly robust and accurate models. Second, the demographics fields were also quite unbalanced from the collected data, which could affect the machine learning results and cause bias in the models towards certain demographics. For example, more female participants than males took part in the online survey; they were mainly young adults aged 18-27. It would be ideal for the data to be gender- and age-balanced. This limitation could be addressed by collecting a large and more balanced dataset across the globe in the future. Third, although the online survey was open to anyone from all over the world, the majority of them came from Asia, North America, and Europe. Due to this uneven distribution, the region of residence in the dataset was loosely categorized into three classes: East, West, and Other. Since the world is much more diverse than three regions, people from different regions of the world could have different levels of anxiety. This problem needs to be addressed by categorizing the regions in a more detailed manner, for example, East Asia, South Asia, Southeast Asia, Western Europe, etc. Fourth, the present study only explored two feature selection techniques: MRMR and Gini Importance in an Extra Tree classifier. To improve the robustness of the most important DASS-42 questions selected, additional supervised and unsupervised feature selection methods, such as the forward feature selection, backward feature elimination, and LASSO regularization, may be tested (Saeys et al., 2007).

### Future Studies

In addition to addressing the above limitations, future studies are needed to test our Long to Short approach further. Because the current study was a proof-of-concept, we only trained models to classify between low vs. high levels of anxiety. In the future, machine learning techniques can be readily used to classify the five severity levels defined in DASS-42: “none”, “mild”, “moderate”, “severe”, and “extremely severe”, allowing trained models to classify between the five levels instead of two. This would require additional data re-balancing and re-sampling of the dataset at each assessment level.

Again, as this is a proof-of-concept study and not meant to be exhaustive, this study only trained models on 10 combinations of 5 DASS questions and 3 demographics fields, or combinations of 7 DASS questions without demographic fields. For greater robustness, we could increase the number of combinations of DASS-42 questions trained to more than 10, so that the results are less biased towards a specific combination of questions. Further, we would be able to obtain more than a 8-question or 7-question version of the shortened assessment of anxiety levels, which would reduce repetition. To this end, we would need to redo our feature selection step to select more than 10 important questions from DASS to have a larger pool of questions to select to form the shortened assessments.

In addition, the current study only examined anxiety levels in the DASS assessment. We could use the same data processing pipeline to predict the levels of depression and stress in DASS as well. This process would simply require us to re-use the pipeline of the present study and train computational models to classify the levels of depression and stress. In addition, due to the nested structure of DASS, we could try shortening each sub-scale and thus shortening the full DASS scale, similar to how the DASS-42 was originally shortened to DASS-21.

Furthermore, the present study used the DASS-42 anxiety score as the ground truth. It is also possible that we could use our data processing pipeline to train the computational models to predict whether an individual has a clinical level of anxiety, stress, or depression using clinical diagnosis by clinicians as the ground truth. To do so, the clinical ground truth data would need to be collected along with the full DASS scale. The resultant computational models would be able to assist rapid screening of patients with anxiety disorder, clinical depression, or stress.

Finally, to extend the present study further, our Long to Short approach could be applied to other questionnaire-based assessments, whether for mental health or for other purposes. A few examples include personality assessments (e.g., the Big Five Personality Traits), standardized ability tests such as IQ tests, aptitude tests such as GRE, MCAT, and LSAT, achievement tests such as SAT, ACT, and TOFEL, and surveys of attitude and opinions (e.g., voting intention, marketing, health, and lifestyle). The implementation of these examples can be completed on a desktop computer, a mobile phone using an app, a web browser, in the cloud and so on.

## Conclusion

This study describes a novel Long to Short approach of using machine learning techniques to develop efficient and convenient short assessments or surveys to approximate a long one. As a proof of concept, this study demonstrates the use of this approach on the Depression Anxiety Stress Scale 42 (DASS-42) scale. We successfully developed a shortened scale of 8 items (5 items from DASS and 3 demographics items – gender, age, and region of residence) and that of 7 items without demographics that predicts anxiety levels (low vs. high) at over 90% accuracy compared to the original 42-item DASS scale. With further development and empirical research, such an approach is promised to be applied to shorten any other assessments used to assess people’s behaviors, opinions, attitudes, mental and physical states, traits, aptitudes, abilities, and mastery of a subject matter with high accuracies.

## Appendix

Table 5

**Table 5:** Default Machine Learning Model Hyperparameters

Model	Python Library and Class	Hyperparameters and Default Values	
Gaussian Naïve Bayes	Scikit-learn sklearn.naive_bayes.GaussianNB Documentation	priors	None (50/50)
		var_smoothing	1e-9
Logistic Regression	Scikit-learn sklearn.linear_model.LogisticRegression Documentation	penalty	l2
		dual	False
		tol (Tolerance for stopping criteria)	1e-4
		C (Inverse of regularization strength)	1
		fit_intercept	True
		intercept_scaling	1
		class_weight	None
		random_state	None
		solver	lbfgs
		max_iter	100
		multi_class	auto
		verbose	0
		warm_start	False
		n_jobs	None
		l1_ratio	None
Support Vector Machine	Scikit-learn sklearn.svm.SVC Documentation	C (Inverse of regularization strength)	1
		kernel	rbf
		degree	3
		gamma	scale
		coef0	0
		shrinking	True
		probability	False
		tol (Tolerance for stopping criteria)	1e-3
		cache_size	200
		class_weight	None
		verbose	False
		max_iter	-1
		decision_function_shape	ovr
		break_ties	False
		random_state	None
Random Forest	Scikit-learn sklearn.ensemble.RandomForestClassifier Documentation	n_estimators	100
		criterion	gini
		max_depth (maximum depth of the tree)	None
		min_samples_split	2
		min_samples_leaf	1
		min_weight_fraction_leaf	0
		max_features	auto
		max_leaf_nodes	None
		min_impurity_decrease	0
		min_impurity_split	None
		bootstrap	True
		oob_score	False
		n_jobs	None
		random_state	None
		verbose	0
		warm_start	False
		class_weight	None
		ccp_alpha	0
		max_samples	None
Multilayer Perceptron	Scikit-learn sklearn.neural_network.MLPClassifier Documentation	hidden_layer_sizes	100
		activation	relu
		solver	adam
		alpha	0.0001
		batch_size	auto
		learning_rate	const
		learning_rate_init	0.001
		max_iter	200
		tol	0.0001
		momentum	0.9
		nesterovs_momentum	True
		beta_1	0.9
		beta_2	0.999
		epsilon	1e-08
XGBoost	XGBoost xgboost.xgbClassifier Documentation	n_estimators	100
		use_label_encoder	False
		max_depth	6
		learning_rate	0.1
		verbosity	0
		objective	linear
		booster	gbtree
		tree_method	auto
		n_jobs	None
		gamma	0
		min_child_weight	1
		max_delta_step	0
		subsample	1
		colsample_bytree	1
		colsample_bylevel	1
		colsample_bynode	1
		reg_alpha	0
		reg_lambda	1
		scale_pos_weight	1
		base_score	0.5
		random_state	0
Stacked Generaliz- ation Ensemble	Scikit-learn sklearn.ensemble.StackingClassifier Documentation Estimators: sklearn.naive_bayes.GaussianNB sklearn.linear_model.LogisticRegression sklearn.svm.SVC sklearn.ensemble.RandomForestClassifier xgboost.xgbClassifier sklearn.neural_network.MLPClassifier (all default hyperparameters) Final classifier: sklearn.linear_model.LogisticRegression	cv (cross validation)	5
		stack_method	predict _proba
		passthrough	False
